# Editorial: Implementation of social and emotional learning interventions in applied settings: approaches to definition, measurement, and analysis

**DOI:** 10.3389/fpsyg.2023.1281083

**Published:** 2023-09-08

**Authors:** Sophie P. Barnes, Celene E. Domitrovich, Stephanie M. Jones

**Affiliations:** ^1^Harvard Graduate School of Education, Harvard University, Cambridge, MA, United States; ^2^Early Childhood Innovation Network, Department of Psychiatry, Georgetown University Medical Center, Washington, DC, United States

**Keywords:** implementation, social and emotional learning (SEL), intervention, social and emotional learning (SEL) skills, measurement, schools

## Implementation matters for SEL intervention effectiveness

More than two decades of meta-analytic research documents the effectiveness of social and emotional learning (SEL) interventions for improving social-emotional competencies and longer-term academic outcomes, behavioral functioning, and mental health (Durlak et al., 2011, 2022; Jones et al., 2021; Cipriano et al., 2023). Implementation research suggests that outcomes are more robust when interventions are implemented with adherence to their intended model (Durlak and DuPre, 2008). In a meta-analysis of 213 studies of SEL interventions, programs implemented with fidelity produced greater improvements in children's outcomes than studies that reported challenges with implementation (Durlak et al., 2011).

There are a number of ways to characterize and measure implementation. Dane and Schneider (1998) defined five dimensions (adherence, exposure, quality, participant responsiveness, and program differentiation) of program integrity that have remained a part of most current definitions of implementation outcomes (Proctor et al., 2011). The term fidelity has emerged over time as a broader term with adherence and dosage as indicators within that dimension (Century et al., 2010; Proctor et al., 2011). Most studies of SEL program implementation assess fidelity or dosage while fewer focus on quality or participant responsiveness (O'Donnell, 2008; Berkel et al., 2011).

Several conceptual frameworks have been developed to illustrate the multiple factors at various ecological levels that influence the implementation process (Wandersman et al., 2008; Damschroder et al., 2009; Meyers et al., 2012). Domitrovich et al. (2008) developed a three-level ecological framework for organizing factors that relate specifically to the implementation of school-based interventions: macro-level factors (e.g., policies and financing, community capacity and empowerment), school-level factors (e.g., organizational functioning, school and classroom culture, and climate), and individual-level factors [e.g., psychological functioning (burnout and self-efficacy) and perceptions of and attitudes toward the intervention]. Several studies have empirically validated the importance of multi-level factors as predictors of SEL implementation (e.g., see Malloy et al., 2015; Domitrovich et al., 2019; Musci et al., 2019; Cramer et al., 2021).

Studies that include fidelity data often report variability at both the individual level between implementers and at the organizational level across the settings (e.g., schools) where programs are delivered, suggesting the need to more deeply understand associations between implementation and outcomes by examining potentially relevant individual, school, and community factors (Durlak and DuPre, 2008). While research underscoring the importance of implementation fidelity for SEL interventions is growing, it is rarely the primary focus of SEL research and is typically not measured or described in sufficient detail. However, we believe that some of what is relatively equivocal in research on SEL programs (e.g., Jones et al., 2019) can be addressed by documenting and understanding implementation with greater precision and depth. In this Research Topic, authors addressed a wide variety of topics including: (1) approaches to measuring SEL implementation and engagement, (2) applying innovative quantitative and qualitative methods, (3) investing in partnerships with practitioners, (4) multi-level factors predicting implementation, (5) equity in defining and measuring SEL and its implementation, and (6) understanding implementation in global contexts (see Table 1 for an overview of all the manuscripts included in this issue). In the sections below we describe key themes and ideas across the many papers in this Research Topic.

**Table 1:** Overview of manuscripts included in the SEL implementation Research Topic.

	**Method**			**Age Group**				**Location**		**Focus**
	**Theoret**.	**Qual**.	**Quant**.	**ECE**	**Elem**.	**M.S./H.S**.	**Adults**	**U.S**.	**Global**	
Bodrova et al.										Theory and predictors
Braun et al.										Theory and predictors
Choles et al.										Measurement and methods
Devlin et al.										Measurement and methods
Dyson et al.										Educator perspectives and partnerships
Gómez et al.										Measurement and methods
Grant et al.										Educator perspectives and partnerships
Harker Roa et al.										Theory and predictors
Hunter et al.										Theory and predictors
Lin et al.										Educator perspectives and partnerships
Martinsone et al.										Measurement and methods
McCoy and Hanno										Theory and predictors
Meland and Brion-Meisels										Measurement and methods
Partee et al.										Educator perspectives and partnerships
Spacciapoli et al.										Educator perspectives and partnerships
Thierry et al.										Theory and predictors
Ulla and Poom-Valickis										Theory and predictors
White et al.										Theory and predictors
Wu et al.										Measurement and methods
Zhao et al.										Measurement and methods

## Measuring SEL implementation and engagement

Two papers described comprehensive implementation measurement strategies that monitored implementation of both the intervention and the support system. Choles et al. developed a two-level conceptual implementation framework and aligned measures to capture fidelity of implementation of a mindfulness-focused SEL program for pre-school children. The model is innovative because it focuses on program implementation supports for teachers (e.g., coaching) as well as teacher implementation of the curriculum in the classroom. Martinsone et al. describe their approach to creating a system for monitoring the implementation of a comprehensive school-based mental health program for elementary and middle school students that included universal curriculum lessons as well as home-based activities. The implementation supports included teacher training and the development of school teams whose members helped ensure program fidelity and quality. The program was delivered in six countries and the authors paid close attention to capturing cultural adaptations as part of fidelity monitoring.

A number of papers proposed new approaches to measuring SEL implementation. Wu et al. designed an approach to capture nuanced features of implementation of non-curricular, flexible approaches organized as brief activities across SEL domains (Mindfulness and Brain Games in this study) in humanitarian settings. The paper presents three dimensions of dosage: quantity (how much), duration (for how long), and temporal pattern (how often). This approach can capture (1) how often activities targeting the same SEL domain are repeated and (2) how many activities are implemented before at least one activity is attempted from each available SEL domain—providing insight into patterns of intervention implementation and exposure, in addition to quantity and duration. Devlin et al. noted that most implementation measures focus on the implementer even though children's engagement during implementation likely influences children's outcomes. The authors developed a four-step protocol designed to capture active child engagement by observing children's behavior. The protocol focused on identifying points of active child engagement, operationalizing and measuring those dimensions, and analyzing the data by linking child engagement to other meaningful variables. Bodrova et al. discuss the importance of play as a context for SEL development during early childhood and the challenges of monitoring the fidelity of this activity when it is a component of an SEL intervention. They argue that play components of SEL interventions need to be made “visible” and that nuanced measures of play need to be developed so research can isolate the important characteristics of play that predict positive social and behavioral development over time. All three papers underscore the need to consider implementation as a multi-faceted, dynamic process that requires attention to multiple aspects of implementation (e.g., implementer and recipient, multiple intervention components, implementation context, etc.).

## Applying innovative quantitative and qualitative methods

Studies employed a variety of innovative qualitative and quantitative analytic approaches. Two studies used latent profile analysis to create descriptive profiles (or categories) of implementation and explore associations between teacher/classroom profiles and children's outcomes. Zhao et al., used measures of dosage, adherence, quality of delivery, and student engagement to identify three latent profiles of implementation (high, moderate, and low). Classrooms with moderate- and high-level implementation practices showed significantly higher gains in student outcomes than those with low-level implementation. Similarly, Gómez et al. identified two latent profiles: below average implementation and above average implementation using measures of teacher responsiveness (teacher evaluation of the training sessions) and amount of exposure to implementation supports (ratings of coaching and time spent with coach). Teachers in the below average profile were less responsive to training and received less support than teachers in the above average profile. Using propensity scores, the authors found that more experienced teachers and teachers reporting lower levels of burnout were more likely to implement the intervention as intended.

Integrating SEL and youth participatory action research (YPAR), a form of critical participatory action research (CPAR), represents another novel and promising methodological approach (Meland and Brion-Meisels). In YPAR, youth are full participants in the research process and seen as the experts of their own lives and contexts. The authors propose a set of four core commitments as the mechanisms of YPAR that nurture SEL (e.g., democratic participation that centers youth expertise) and conceptualize SEL implementation integrity as adherence to a set of commitments rather than fidelity to a specific set of activities. This approach can provide more flexible ways to think about implementation that centers youth empowerment and voice.

We were also pleased to receive several papers using qualitative methods that focus on understanding individual perceptions and experiences. Using interviews, focus groups, and observations, Dyson et al. explore educators' views on SEL in a rural, high-needs elementary school setting. While educators “bought-in” to SEL, they reported lack of time, lack of preparation and development, home-school disconnection, and pushback from students as significant constraints. Another study used a mixed-methods approach to study the Early Childhood Mental Health Consultation pilot in Virginia. Partee et al. interviewed participants who chose not to participate in ECMHC or opted out after consultation began and conducted focus groups with participants who had sustained engagement. These qualitative papers provide perspectives from a wider set of voices often not included in traditional impact and implementation research including rural settings and those who opt *not* to participate in interventions.

## Investing in partnerships with practitioners

Building on the themes of incorporating previously undervalued voices in new ways, a handful of studies centered partnerships with educators. Grant et al. shared findings from a multi-year research-practice partnership (RPP) designed to support SEL implementation in a district. The authors offer key lessons learned related to developing feasible and meaningful implementation measures, identifying structures that can support the collection and use of implementation data to improve practice, presenting data for various audiences, and creating systems for sustainable data use. Spacciapoli et al. conceptualize fidelity as part of an ongoing professional development feedback ecosystem. Teachers record short videos across the school year, review and reflect on their video, and receive targeted feedback from a coach. The method approaches fidelity of implementation as a developmental journey with the expectation that teachers will improve over time and develop a nuanced set of indicators across the school year. These examples demonstrate that mutually beneficial relationships between researchers, practitioners, and other stakeholders can create conditions for iterative cycles of design and testing and the development of sustainable systems of SEL implementation, data collection, and use.

## Multi-level factors predicting implementation

A number of studies examined predictors of implementation. Ulla and Poom-Valickis conducted a systematic review and identified four categories of contextual factors that can influence implementation: program support, school, teacher, and student level factors. Their analysis focused on the relative importance of these factors and found that the most frequent statistically significant factors included modeling activities during coaching and teacher-coach working relationship. Teacher burnout was uniquely related to program dosage. In community-based childcare centers, Hunter et al. examined factors that predict the implementation of a comprehensive pre-school program that includes curricular components and teaching practices designed to promote the social-emotional development of young children. They found that baseline teaching practices and responsiveness to intervention (and not teacher education or experience) predicted quality of intervention activities and teaching strategy delivery. Workplace factors (e.g., classroom resources and job satisfaction) predicted multiple features of implementation. Braun et al. also examined workplace factors (i.e., occupational health) that predict the implementation of a universal social-emotional learning program implemented in elementary schools by early career teachers. The authors went a step beyond typical research examining the main effects of implementation predictors to test interactions among factors. They found perceptions of program feasibility moderated the relationship between job stress and implementation quality in unexpected ways. In a unique study of implementation predictors, Thierry et al. conducted secondary analyses of a national dataset that included information on school district and macro-level factors to explore factors associated with teacher and counselor-facilitated delivery of a universal social-emotional learning program conducted in elementary and middle schools. The studies included in this section highlight the multi-level factors that shape the conditions for SEL implementation in schools.

## Equity in defining and measuring SEL and its implementation

Three papers included equity as a central component. Lin et al. examined how pre-service educators define SEL. Educators conceptualized SEL as broader than competency-based models. They instead considered SEL as an opportunity to respond to student and community needs, center humanity, and advance social justice. Participants advocated for a co-created, humanizing SEL approach that honors identity, promotes justice, and ultimately can dismantle inequitable systems. YPAR, the approach described by Meland and Brion-Meisels, elevates individual voices and empowers youth to engage in collective action that aims to disrupt systems of inequity and promote positive change in communities. Underlying the approach is a trusting, equitable, reciprocal relationship that remains a central part of the entire YPAR process. Finally, Spacciapoli et al. noted that measures of implementation often leave teachers “in the dark” about observation goals and items as well as implementation strengths and areas for improvement. The authors' transparent approach includes teachers as collaborators in implementation measurement and tracking by engaging them in observing and reflecting on their practice in order to create equitable partnerships.

## Understanding implementation in global contexts

The focus of papers in this Research Topic describe SEL efforts across the globe including programming that was delivered in humanitarian settings in Sierra Leone (Wu et al.), community-based programs conducted in Rio de Janeiro, Brazil (McCoy and Hanno), and Colombia (Harker Roa et al.), and a comprehensive school-based mental health program implemented in several European countries (Italy, Latvia, Romania, Croatia, Greece, and Portugal; Martinsone et al.). The two papers describing SEL efforts in South America both discuss the challenges associated with delivering programs in extreme conditions including community violence, forced displacement, and extreme adversity and the importance of cultural adaptation and flexibility. Harker Roa et al. identified cultural adaptation as an implementation enabler that was successful when it was conducted intentionally following a structured protocol. They also found that with sufficient training and support, their parent-focused program could be delivered by paraprofessionals, an example of “task-shifting” that was necessary because of the shortage of mental health professionals in Colombia. McCoy and Hanno also identify culture as a key factor that influenced the delivery of their SEL programming in Brazil. Their perspectives on the importance of this and other macro-level factors including timing and government support came from their work delivering an SEL program in elementary schools and from their review of similar research conducted in low-income, conflict affected settings.

## Considerations for the future

Put quite simply, the collection of articles in this Research Topic tell us unequivocally that the quality and quantity of implementation of social and emotional learning programs, strategies, and practices is the cornerstone to their efficacy. But that is not all these innovative and penetrating articles tell us. Indeed, they go beyond relatively “simple” questions of whether and how implementation factors make a difference and stretch our body of knowledge, pushing us to (1) consider new ways of measuring, operationalizing, and analyzing implementation data, (2) incorporate the perspectives of those often not represented in our implementation data, (3) expand our view well beyond our own borders and bring multiple contexts and settings into the broader body of work, and (4) sustain SEL by developing innovative support systems that address both individual and contextual factors that influence the process of implementation. In addition, these papers leave us with some directions to consider for future work. Among many possible directions for future research, we highlight three key questions that build from these papers and, in our view, are central to moving the field forward.

- What are the common and unique, multi-level, predictors of implementation quality and quantity that ultimately represent universal- and program-specific factors?
- Can we embed implementation data collection, reflection, and adapted practice into program design and delivery in ways that create meaningful improvements, rather than considering implementation as an added and separate effort?
- What do cross-cutting and persistent patterns of implementation (i.e., lower than expected dosage, fidelity, and overall engagement) suggest about potential changes required to program design, delivery expectations, and pre-implementation training?

## Author contributions

SB: Writing—original draft, Writing—review and editing. CD: Writing—review and editing. SJ: Writing—review and editing.
